# Editorial: Insights in Clinical Microbiology: 2021

**DOI:** 10.3389/fcimb.2022.928344

**Published:** 2022-05-25

**Authors:** Max Maurin

**Affiliations:** Univ. Grenoble Alpes, Centre National de la Recherche Scientifique (CNRS), Grenoble Institut National Polytechnique (INP), Centre Hospitalier Universitaire (CHU) Grenoble Alpes, Techniques de l’imagerie, Modélisation et Cognition (TIMC), Grenoble, France

**Keywords:** COVID-19, antibiotic resistance, pneumonia, immunocompromised patient, transplanted, non-Hodgkin lymphoma, periodontitis

The broad spectrum of the topic “Insights In Clinical Microbiology: 2021” was intended to allow the submission of publications on various themes, while knowing that a limited part of the scientific fields would be represented.

SARS-CoV-2 (severe acute respiratory syndrome Coronavirus 2) has caused a pandemic of COVID-19 (Coronavirus disase-2019). As of 22 April 2022, the WHO estimates that about 505 millions of COVID-19 confirmed cases have occurred worldwide since the start of the pandemic, causing about 6.2 millions deaths (Coronavirus disease (COVID-19) Situation Dashboard; https://covid19.who.int/).

Escobedo et al. highlighted recent knowledge on COVID-19. SARS-CoV-2 is an RNA virus closely related to the bat CoV RaTG13, suggesting bats are the origin of SARS-Cov-2. No intermediate host has been formally identified. SARS-Cov-2 major clades and variants have been characterized, the latter being classified as variants of interest (VOIs), variants of concern (VOCs), and variants of high consequence (VHCs). COVID-19 can manifest as an acute, subacute or chronic disease. It can lead to severe life-threatening complications involving variable organs (e.g., the lungs, the heart, and the brain). Diagnosis is based on molecular tests. Anti-SARS-CoV-2 treatment options mainly include dexamethasone, antivirals and monoclonal antibodies. An effective and well-tolerated treatment that could be used at different stages of the disease remains to be developed. Vaccines are effective to prevent severe forms of COVID-19. Mutations affecting the SARS-CoV-2 spike protein could alter their effectiveness. The finding of risk factors associated with COVID-19 complications (e.g., the host innate antiviral defense and the host-driven inflammatory lung injury) could lead to personalized treatment.

Hong et al. developed machine learning models and a nomogram to differentiate critically ill (CI) from non-critically ill (non-CI) COVID-19 pneumonia patients. Using a logistic regression function (LR), Random Forest, and XGBoost models, statistically significant differences between CI and non-CI included were found for serum levels of interleukin-6, interleukin (IL)-10, T cells, CD4+ T, and CD8+ T cells. IL-10 was the most useful predictor of CI patients with COVID-19 pneumonia. XGBoost exhibited the highest discriminatory performance. There results could help better management of COVID-19 pneumonia patients.

Antibiotic resistance in human and animal bacterial pathogens remains a challenging issue. Most newly developed compounds belong to known antibiotic classes. Development of bacterial resistances to these drugs may be fast and should be carefully monitored.

Lazzaro et al. describe the impact of alterations in the penicillin-binding protein 4 (PBP4) on susceptibility of *Enterococcus faecalis* to ceftobiprole, a newly developed fifth-generation cephalosporin. These authors demonstrate that mutations affecting the DNA sequence of the PBP4 gene can increase its expression level and alter the catalytic site of PBP4 leading to reduced susceptibility of *E. faecalis* clinical isolates to ceftobiprole. Apart from revealing the mechanism of ceftobiprole non-susceptibility in *E. faecalis* strains, this study encourages monitoring bacterial resistance to this cephalosporin.

*Mycoplasma pneumoniae* can cause atypical bacterial pneumonia, which may occasionally evolve to severe pulmonary or extrapulmonary complications. Predicting such evolution remains challenging. Zhou et al. evaluated 275 children infected with *M. pneumoniae*, including 147 with complication and 128 without. Patients with complications of necrotizing pneumonitis, pneumothorax, skin rash, or bronchiolitis obliterans had higher IgE levels (p = 0.041), which could then be considered a potential biomarker for severe *M. pneumoniae* pneumonia.

Diagnosis of infections in the immunocompromised patient is challenging because of a broad spectrum of potential pathogens. Defining the true infectious risk is thus essential to implement appropriate management of these patients.

Organ transplantation exposes the transplanted patient receiving immunosuppressive treatment to severe infections. Yu et al. evaluated donor-derived B19V infection in patients receiving a kidney transplant, either from a living donor (LD, n=823) or a deceased donor (DD, 1,225). B19V infection was diagnosed through quantification of B19V-DNA in serum by real-time PCR. The incidence of B19V DNAemia was 0.4% in recipients of LD transplants and 1.5% in recipients of DD transplant. The authors concluded that routine screening for B19V in donors seems unnecessary and a kidney transplant from a B19V donor can be considered acceptable.

Pulmonary infections in non-Hodgkin’s lymphoma (NHL) patients are frequent and difficult to diagnose. Zhang et al. evaluated the cytokine profiles of 229 NHL patients to tentatively predict respiratory bacterial infection and bacteremia. They found that cytokine levels, including interleukin-6 (IL-6), IL-8, IL-10,TNF-beta, and IFN-gamma, were much higher in NHL patients with respiratory bacterial infections then in those without infection. As for IL-6, levels ≥18.79pg/mL were associated with pulmonary bacterial infection and levels ≥102.6pg/mL with pulmonary infection and bacteremia. The authors suggested that cytokines levels could help diagnose pulmonary bacterial infection in NHL patients.

According to a Global Burden of Diseases study (2019), periodontal disease affects 20–50% of the worldwide population (https://doi.org/10.1016/S0140-6736(19)31146-8). However, there is a high disparity in periodontitis prevalence between different populations. Wang et al. evaluated potential microbiological risk factors associated with periodontitis severity and disparities by qPCR testing of dental plaque samples from 67 Caucasians Americans (CAs), 56 African Americans (AAs), and 68 Hispanic Americans (HAs). AAs had a significantly higher bacterial mass than the CAs and HAs. Much higher levels of *Porphyromonas gingivalis* (a periodontitis-associated bacterium) were found in the AAs and HAs compared to CAs. The ratio of *Streptococcus cristatus* to *P. gingivalis* was significantly higher in CAs than in HAs and AAs, suggesting a protective role of the former. The authors concluded that variations in the dental plaque microbiota could be risk factors of disparities and severity of periodontal disease.

As editor of the **“Insights In Clinical Microbiology: 2021”** Research Topic, I would like to acknowledge all contributing authors for providing recent advances in their fields of research. Knowledge in clinical microbiology is constantly progressing, allowing better management of infectious diseases.

## Author Contributions

The author confirms being the sole contributor of this work and has approved it for publication.

## Conflict of Interest

The author declares that the research was conducted in the absence of any commercial or financial relationships that could be construed as a potential conflict of interest.

## Publisher’s Note

All claims expressed in this article are solely those of the authors and do not necessarily represent those of their affiliated organizations, or those of the publisher, the editors and the reviewers. Any product that may be evaluated in this article, or claim that may be made by its manufacturer, is not guaranteed or endorsed by the publisher.

